# The development of functional opsonophagocytic assays to evaluate antibody responses to *Klebsiella pneumoniae* capsular antigens

**DOI:** 10.1128/msphere.00176-25

**Published:** 2025-06-12

**Authors:** Robert Lawrence, Emma Bownes, Marina Johnson, Heather Fox, Drew Huff, Ivan Olave, Anup Datta, David Goldblatt, Nathalie Karaky

**Affiliations:** 1 Great Ormond Street Institute of Child Health, University College London 4919, London, United Kingdom; 2 Inventprise, Inc. 706260, Redmond, Washington, USA; University of Maryland School of Medicine, Baltimore, Maryland, USA

**Keywords:** *Klebsiella pneumoniae*, vaccines, opsonophagocytic killing assay, K antigen, capsule

## Abstract

*Klebsiella pneumoniae* is one of the leading causes of nosocomial infections in low- and middle-income countries (LMICs), with a high mortality rate among the immunocompromised. With increasing antibiotic resistance, there is an urgent need for preventive measures such as vaccines, but none are currently licensed for use. In order to evaluate natural immunity and assess the immunogenicity of novel vaccines, we set out to develop functional assays that effectively measure the immune response of *K. pneumoniae* anti-capsular antibodies *in vitro*. Serotypes KL2, KL15, KL25, KL62, and KL102 were targeted as these are five of the most prevalent and invasive strains, particularly in LMIC settings, and are putative vaccine antigens. Opsonophagocytic killing assays (OPAs) for each serotype were developed and qualified. Serotype-specific IgG from vaccinated rabbit sera and human sera was used to demonstrate *in vitro* antibody and complement-mediated killing for all serotypes tested, whereas cross-reactivity between each serotype was minimal by competitive analyses. These assays act as a platform to allow further serological evaluation of natural immunity and the performance of *K. pneumoniae* vaccines. Understanding the function of vaccine-induced antibodies, as well as natural IgG induced by exposure to *K. pneumoniae*, will be crucial to determine correlates of protection and aid in the path to licensure of a *K. pneumoniae* vaccine.

**IMPORTANCE:**

*K. pneumoniae* is a pathogen that causes serious infections such as pneumonia and sepsis globally. The increasing prevalence of antibiotic resistance in this pathogen has complicated treatment efforts, highlighting the need for preventive therapeutic strategies such as vaccination. However, no licensed vaccines are currently available. Standardized assays to assess the immunogenicity of new vaccines are crucial for vaccine development and evaluation of other therapeutics. Therefore, we have developed assays that can assess the functionality of antibodies, which can be used to evaluate the potential of novel *K. pneumoniae* conjugate vaccines, and inform which antibodies are most effective for preventing disease.

## INTRODUCTION

*K. pneumoniae* is a gram-negative, encapsulated, opportunistic pathogen that commonly colonizes human mucosal surfaces (1). As one of the ESKAPE pathogens, *K. pneumoniae* represents a significant threat in the fight against antimicrobial resistance (AMR). By acquiring a diverse range of β-lactamase enzymes such as extended-spectrum β-lactamases and carbapenemases, *K. pneumoniae* can hydrolyze the β-lactam ring component of penicillin and cephalosporins, making them resistant to many common antibiotic treatments (2, 3). Additionally, the acquisition of plasmids containing genes that enhance capsule production and encode additional siderophores leads to a hypervirulent *K. pneumoniae* (hvKp) pathotype that is capable of infecting young, healthy individuals in a community setting (4, 5). This combination of widespread antibiotic resistance and the prevalence of hypervirulent strains worldwide has created an urgent need for effective strategies to combat this pathogen. Consequently, the World Health Organization (WHO) classified *K. pneumoniae* as a critical pathogen for which new treatments or preventive measures are urgently required (6).

One preventive measure of high priority is the development of a vaccine to prevent *K. pneumoniae* disease. *K. pneumoniae* has two prominent virulence factors in the lipopolysaccharide (LPS) and capsular polysaccharide (CPS), both potentially amenable to use as vaccine constituents. The CPS (K antigen) is a polysaccharide matrix of four to six repeating sugar units that protect the bacteria against host immune responses, such as phagocytosis and complement-mediated killing (5). Use of capsular polysaccharides as vaccine antigens has been a highly successful strategy for bacteria such as *Haemophilus influenzae* type b, *Neisseria meningitidis,* and *Streptococcus pneumoniae* (7–9) and is one strategy being considered for a *K. pneumoniae* vaccine. Recent genomic analyses of over 2,500 *K*. *pneumoniae* isolates have identified more than 150 distinct CPS structures based on unique K loci (known as KL types) (10, 11). This extensive diversity of serotypes presents a significant challenge for their use as vaccine targets, although epidemiological studies have shown that only 25 KL-types are responsible for ~70% of *K. pneumoniae* bacteraemia infections (12, 13), suggesting a capsular polysaccharide vaccine may be a viable vaccine strategy. In contrast, the LPS (comprising a terminal O antigen) has only 11 O serotypes, with additional sub-serotypes that are often cross-reactive, also making it a promising target for vaccines (14–16). However, recent studies have shown that certain K types were able to occlude the LPS and diminish O-antibody binding, which was highlighted by using a capsule-deficient mutant strain (17). Heavily encapsulated strains might therefore demonstrate O antigen masking, reducing the effectiveness of a vaccine based solely on immune responses to O antigen. Thus, although more varied in target antigens, the use of CPS in vaccines is a promising approach.

To help develop and evaluate *K. pneumoniae* vaccines, sensitive and specific immunological assays are required. Immunological assays that have proved most useful for other bacterial capsular polysaccharide-based vaccines have been IgG binding assays combined with functional serum bactericidal (SBAs) or opsonophagocytic assays (OPAs) (18). Such assays have also been used to address potential correlates of protection, providing an alternative route to licensure based on safety and immunological data alone (19). SBAs for the study of *K. pneumoniae* have been successful in proving the differences in activity between antibodies targeting the K and O antigens (17, 20), but given *K. pneumoniae* has a thick capsular polysaccharide layer to evade killing by serum complement (21), and the importance of phagocytic cells in the adaptive immune response in humans, an OPA utilizing an HL-60 cell line is preferable for vaccine evaluation. Therefore, we have developed and qualified OPAs to measure *K. pneumoniae* anti-capsular antibody function. This work is based on our previous experience in utilizing these assays for *S. pneumoniae* vaccine development (22), as well as for the O antigen of *K. pneumoniae* (23). For this study, assays were adapted for five highly prevalent KL types of *K. pneumoniae*: KL2, KL15, KL25, KL62, and KL102 (24, 25). These assays are not only important for accurately determining the immunogenicity of novel vaccines but also allow us to measure the functional or protective effect of antibodies generated during natural infections. This will help inform which surface antigen targets are optimal for future vaccine design and may also pave the way for determining correlates of protection by highlighting the threshold levels of functional serotype-specific IgG needed to prevent infection.

## RESULTS

### Strain selection and assay optimization

**Fig 1:**
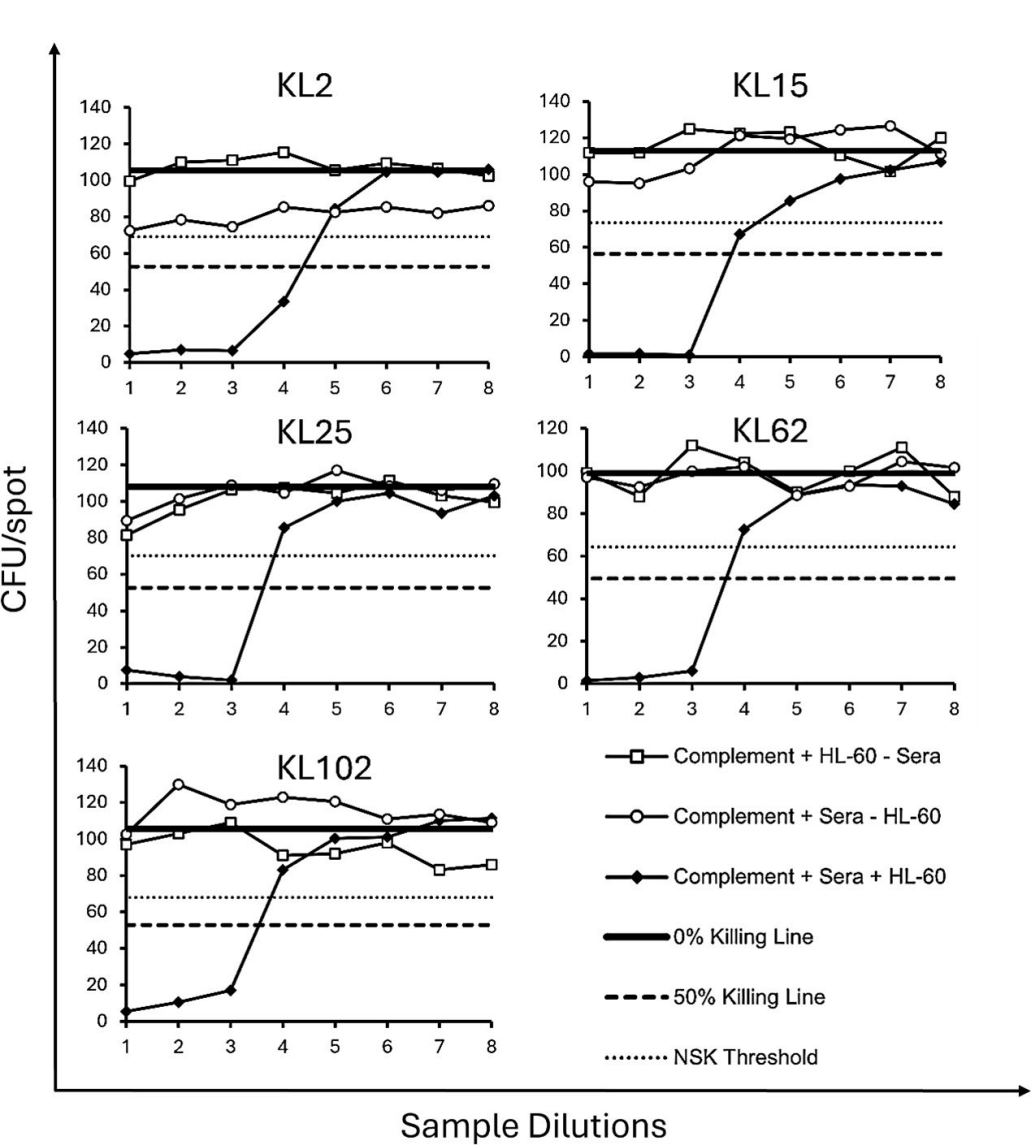
OPA setup and optimization. Strains of each serotype were run in the presence of complement and HL-60 cells, but in the absence of plasma to determine the percent NSK. Strains were also run with plasma (H-IVIG) in the presence and absence of HL-60 cells to determine whether strains could be run as SBAs. H-IVIG was serially diluted eight times (*x*-axis), and the CFU per spot were counted (*y*-axis). Graphs show the strains selected due to low levels of NSK (<35% threshold) and moderate levels of killing with plasma in the presence of cells. NSK = non-specific killing, SBA = serum bactericidal assay, and H-IVIG = hyperimmune globulins for intravenous use.

To identify suitable strains for use in the assays, a collection of clinical culture-confirmed *K. pneumoniae* strains of each serotype were tested for susceptibility to killing *in vitro* by sera/plasma positive for *K. pneumoniae* anti-capsular antibodies. Sera included pooled post-vaccination rabbit sera and human normal subcutaneous immunoglobulin (SCIg), whereas plasma was hyperimmune globulins for intravenous use (H-IVIG), a pooled sample from donors who received one dose of a 24-valent capsular polysaccharide vaccine in the early 1990s (26). Various strains of each serotype were first screened in an optical density (OD1) assay, where a range of concentrations of active baby rabbit complement (BRC) was assessed to determine the level of non-specific killing (NSK), which represents killing attributable to complement alone in the absence of sera/plasma. Strains yielding >35% NSK or with colony-forming units (CFU) < 50 were excluded from further assay optimization (Table S1). Strains with <35% NSK were then assessed by OD2 assay, following the conditions optimized from the OD1, including the addition of H-IVIG as plasma (Fig. 1). The strains that were selected were those that demonstrated intermediate killing and maintained a low percent NSK. The strains were also run in the absence of phagocytic HL-60 cells to determine whether cells are necessary for bacterial killing *in vitro* (Fig. 1). All the strains selected for further qualification required the presence of HL-60 cells to kill; therefore, all assays were developed as OPAs. The target strains (along with their “backbone” antigens) were KL2 (O1), KL15 (O4), KL25 (O1), KL62 (O1), and KL102 (O2afg) (Table 1). Since each strain expresses both a K antigen and an O antigen, to ensure that the killing measured in the assay was attributable to specific anti-K antibodies, we performed specificity assays for each strain.

**TABLE 1:** *K. pneumoniae* strains qualified for use in the OPA^*a*^

Strain ID	Source	KL type	O type
42070_1#108	QECH, Malawi	KL2	O1
20LM-121M0145	GOSH, UK	KL15	O4
i200-21	SSI, Denmark	KL25	O1
i165-21	SSI, Denmark	KL62	O1
42092_2#56	QECH, Malawi	KL102	O2afg

^*a*^ QECH = Queen Elizabeth Central Hospital, SSI = Statens Serum Institut, GOSH = Great Ormond Street Hospital.

### Specificity

Specificity was assessed by competitive analyses, where the amount of inhibition caused by pre-adsorbing H-IVIG with either homologous- or heterologous-purified polysaccharide antigens was assessed. There was high homologous inhibition among all serotypes; pre-adsorption with homologous polysaccharides reduced the opsonic index (OI) by >99% (Table 2). There was minimal (<15%) heterologous capsular inhibition for capsules KL2, KL15, KL25, and KL62 (Table 2). KL102 did, however, demonstrate some cross-reactivity with KL15 and KL62, respectively, perhaps due to molecular mimicry. There was minimal inhibition (<15%) from the relevant O antigen for capsules KL25, KL62, and KL102. However, adsorption with purified O1-OPS reduced the killing of KL2 by 42% and adsorption with O4-OPS reduced the killing of KL15 by 27%. As adsorption with homologous capsules for these two serotypes completely abrogated killing, it is likely that capsular antigen-mediated killing is more dominant, and thus, the assay remains useful for the purpose of exploring immunity.

**TABLE 2:** OPA specificity^*a*^

Antigen	% inhibition				
	KL2:O1	KL15:O4	KL25:O1	KL62:O1	KL102:O2afg
KL2-CPS	99.6	12.5	9.9	9.3	0.0
KL15-CPS	0.9	99.8	14.9	10.9	37.1
KL25-CPS	2.0	7.9	99.8	0.0	0.0
KL62-CPS	0.0	11.4	10.2	99.5	25.7
KL102-CPS	0.0	12.3	4.4	−3.7	99.6
Relevant OPS	41.8	27.0	3.2	13.5	3.1

^*a*^ Plasma (H-IVIG) was pre-adsorbed with 1 µg/mL of purified polysaccharide antigens for > 60 min, and the OI was compared with a no competitor control diluted in assay buffer alone. Homologous antigen inhibition is highlighted by grey cells. Only the relevant O antigen for each strain was used, that is, in the KL15 OPA, O4-OPS was used. % inhibition was calculated as 100-(OI with competitor/OI without competitor)*100. OPA = opsonophagocytic assay, OI = opsonic index, CPS = capsular polysaccharide, OPS = O antigen polysaccharide, and H-IVIG = hyperimmune globulins for intravenous use.

### Precision

Assay precision and repeatability were determined by running pooled, post-vaccination sera/plasma, including rabbit sera (pools 1–3) and H-IVIG (pool 4), on one plate over 3 non-consecutive days by different technicians (inter-assay precision), and on three plates on the same day (intra-assay precision). The average coefficient of variation (%CV) was 15.4% across all serotypes in the inter-assay and 10.8% in the intra-assay (Table 3). The resulting OI of each pooled sample was also within one 3-fold variation of the mean for all serotypes, suggesting good assay precision.

**TABLE 3:** OPA precision^*a*^

Serotype	Inter-assay CV (%)				Intra-assay CV (%)			
	Pool 1	Pool 2	Pool 3	Pool 4	Pool 1	Pool 2	Pool 3	Pool 4
KL2	8.3	11.7	10.9	16.2	8.7	3.4	2.5	3.2
KL15	N/A	N/A	N/A	15.3	N/A	N/A	N/A	11.1
KL25	7.3	10.3	6.4	17.4	10.4	9.1	1.9	5.2
KL62	N/A	N/A	N/A	14.4	N/A	N/A	N/A	11.4
KL102	17.8	30.6	22.1	30.2	6.7	41.4	25.7	8.1

^*a*^ Mean %CV is calculated from three replicate experiments. Samples used for the KL2, KL25, and KL102 OPAs were pooled on day 42 post-vaccination rabbit sera (pools 1-3; *n* = 3) and post-vaccination human plasma H-IVIG (pool 4; *n* = 3). For KL15 and KL62 OPAs, only H-IVIG (pool 4; *n* = 12) was used as the rabbit sera did not contain the appropriate IgG to cause killing. CV = coefficient of variation, OPA = opsonophagocytic assay, H-IVIG = hyperimmune globulins for intravenous use.

### Dilutability and linearity

**Fig 2:**
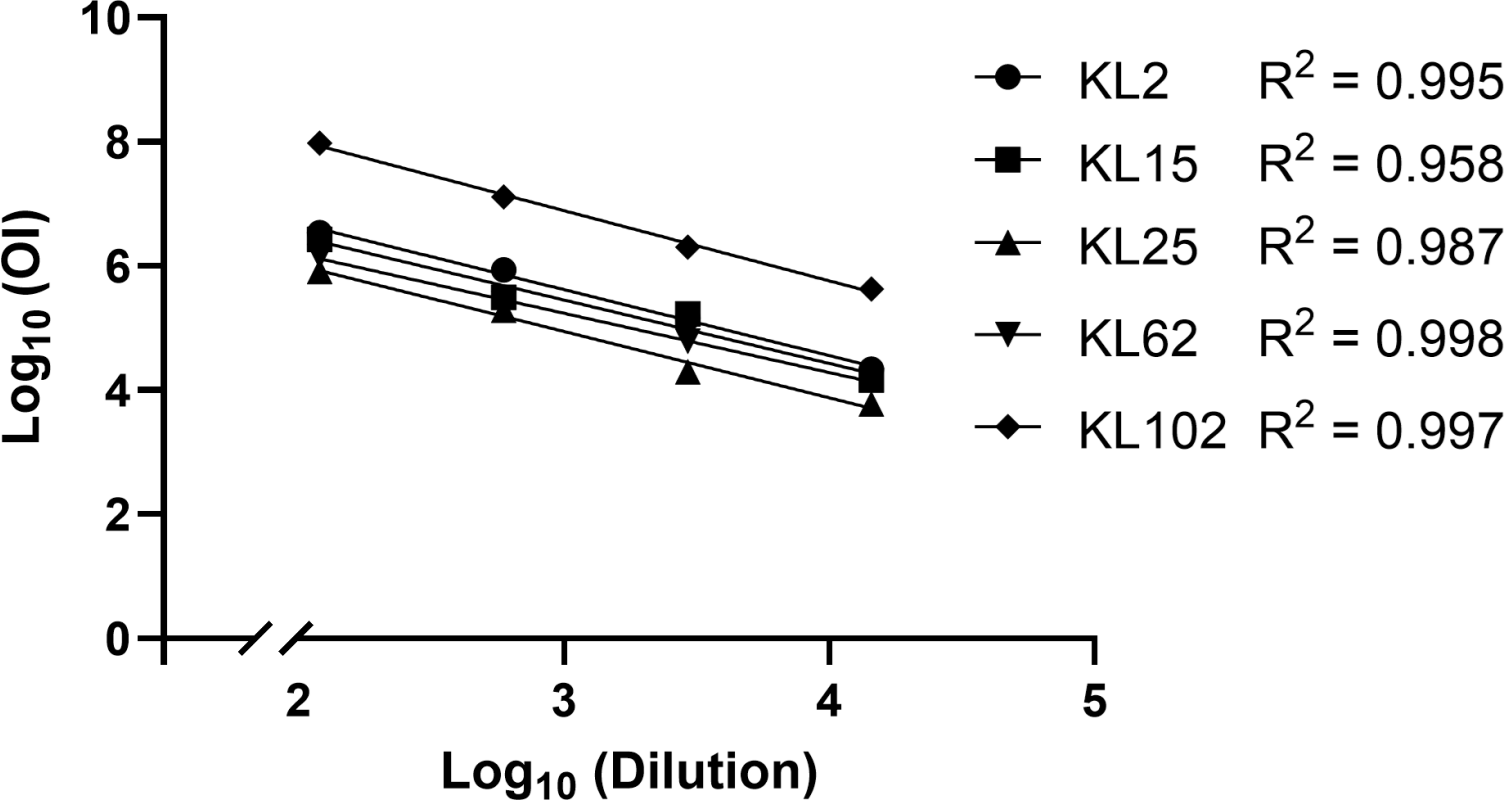
Linearity of KL2, KL15, KL25, KL62, and KL102. H-IVIG was serially diluted 1:8, 1:16, 1:32, and 1:64, and run on each OPA. Log-transformed dilutions (*x*-axis) were plotted against log-transformed OI titers (*y*-axis), and the slope of the regression line and *R*^2^ value were calculated by linear regression. H-IVIG = hyperimmune globulins for intravenous use, OI = opsonic index.

Dilutability was measured by serially diluting a positive sample and calculating the %CV of each result from the mean OI. The %CV values were 10.5%, 16.7%, 19.5%, 16.9%, and 13.9%, for KL2, KL15, KL25, KL62, and KL102, respectively (Table 4). The results at every dilution were also 100% agreeable, all falling within a 3-fold variation of the mean (Table 4). Linearity was determined by spiking positive sera at four different concentrations in antibody-depleted human sera, and the correlation between the OI and dilution was assessed by linear regression (Fig. 2). A Pearson’s correlation coefficient (*R*^2^) value of >0.95 was required to be suitable for the OPA assay. The *R*^2^ values for KL2, KL15, KL25, KL62, and KL102 were 0.995, 0.958, 0.987, 0.998, and 0.997, respectively (Table 4).

**TABLE 4:** OPA qualification summary^*a*^

Parameter	Means of assessment	KL2	KL15	KL25	KL62	KL102
Precision	Inter-assay CV (%)	11.8	15.3	10.4	14.4	25.2
	Intra-assay CV (%)	4.5	11.1	6.6	11.4	20.5
Dilutability	Agreement (%)	100	100	100	100	100
	CV (%)	10.5	16.7	19.5	16.9	13.9
Linearity	*R*²	0.995	0.958	0.987	0.998	0.997
	Slope	−1.062	−1.021	−1.067	−0.954	−1.131
LLOQ	OI	8	6	7	8	9

^*a*^ OPA = opsonophagocytic assay, CV = coefficient of variation, LLOQ = lower limit of quantification, OI = opsonic index.

### LLOQ

The lower limit of quantification (LLOQ) was determined for each serotype by spiking antibody-depleted (non-immune) human sera with low concentrations of positive sera. This was repeated over 3 non-consecutive days, and the median OI result obtained where the maximum killing of colony-forming units (CFU) was ≥70%, where ≥ 80% of the results were within one 3-fold change of the median titer was defined as the LLOQ. This value represents the lowest positive result that can reliably be measured by the OPA and was determined as an OI titer of eight for KL2 and KL62, six for KL15, seven for KL25, and nine for KL102 (Table 4). As the initial dilution of samples can be increased before addition to the plates, there is no functional upper limit of the assay.

## DISCUSSION

Vaccines against *K. pneumoniae* are urgently needed in light of the limited therapeutic options following the increasing threat of AMR and the prevalence of hypervirulent strains worldwide. A better understanding of the immunology of *K. pneumoniae* would also help direct the design and evaluation of putative *K. pneumoniae* vaccines. We therefore developed and qualified OPAs to evaluate antibody function directed at *K. pneumoniae* K antigens, having previously established assays for O antigens (23). The use of quality-controlled reagents that are batch-tested was crucial for the qualification of these assays, given the inherent variability of using biological components, such as baby rabbit complement and HL-60 cells. During assay optimization, KL2, KL15, KL25, KL62, and KL102 strains were tested in the OPA with and without phagocytic HL-60 cells, and all required the presence of these cells to cause significant bacterial killing. This suggests these capsular polysaccharides are able to prevent either C3b deposition or membrane attack complex (MAC) formation on the cell surface due to their thick capsules, as others have described (27, 28). We therefore opted to run each assay as an OPA, rather than an SBA, to allow for measurable bacterial killing and better match the human immune response.

Qualification of the OPA for each serotype included assay precision, dilutability, linearity, LLOQ, and specificity. To assess accuracy, samples with assigned values are needed. As such, reference standards for *K. pneumoniae* have not yet been developed, relative accuracy (also called dilutability) was evaluated instead. Dilutability and linearity are attributes of a biological assay that demonstrate that as a test sample is serially diluted, the results obtained are equivalent once the dilution is accounted for. Assay dilutability was achieved with <20% CV across multiple dilutions for each serotype, and high linearity for all OPAs was also observed, with an average *R*^2^ value of 0.987 across all serotypes. The OPAs also performed reliably with mean inter-assay variation ranging from 10.4% to 25.2% and mean intra-assay variation ranging from 4.5% to 20.5%. This level of variation is in line with other groups’ OPAs of <30%, based on the guidelines set up by the University of Alabama Birmingham protocol (29–31).

Specificity for each serotype was determined in the OPA by pre-adsorbing positive sera with homologous and heterologous antigens. The OPAs demonstrated a high degree of homologous specificity for each target antigen, with near total abrogation of killing and over 99% inhibition in the OI for all serotypes. Although the KL2, KL25, and KL62 strains all express an O1 LPS on their surface, the relative contribution of killing from O1 differed between the strains. There was relatively little O1-mediated killing of KL25 and KL62, but when H-IVIG was adsorbed with purified O1-OPS, there was a 1.7-fold decrease in the killing of the KL2 strain. Clearly, a small amount of immunity is directed to the O1-expressed antigen on this strain, but the complete abrogation of killing when adsorbing with purified KL2 suggests that the K capsule is the dominant antigen. Antibodies targeting the LPS may be less effective at opsonizing and killing *K. pneumoniae* due to its thick capsule, which has been previously shown both *in vitro* and *in vivo* in mice (17, 32). It therefore seems that a vaccine targeting LPS alone may be inadequate at providing sufficient protection and that a combination of K and O serotypes may be the most optimal to achieve a wider coverage and prevent disease.

OPAs were optimized and qualified for measuring antibody function to evaluate natural immunity and the immunogenicity of novel *K. pneumoniae* vaccines. However, this study had several limitations. Although the assays we have established are qualified, we have no way of assessing how they compare with other assays in the public domain and therefore cannot compare results obtained in studies using our assays to others. One step toward achieving this would be to have an established reference standard for accurate assignment of OPA titers, but agreement on an assay for general use and subsequent standardization between laboratories of assays would be required (33). Second, the precision analysis was based on data from measurements conducted over a relatively short time frame within a single laboratory setting. Further analysis over a longer time period would help better elucidate the precision of these assays, particularly using different lots of complement and bacteria, as they could be a source of high variation. Finally, the assays are developed using a single strain that meets all the criteria required for an individual capsule-based assay. How representative the strain chosen for the assay is of all strains with identical capsules is not clear, but it is also not a priority for this stage of the work. We have sought to develop assays that represent killing mediated by the antigen of interest that can be used to evaluate natural, but more importantly, vaccine-specific immunity. Whether the strains chosen are representative of all similarly encapsulated strains can only be evaluated with sera from a significant number of vaccinees, which can be applied to large collections of relevant clinical strains. That is our intention once human phase I studies have been completed. Future work in our lab will also focus on the development of additional assays targeting prevalent K and O serotypes.

There are currently no immune correlates of protection for invasive *K. pneumoniae* disease to infer vaccine efficacy or protection, making licensure of vaccines more difficult (19). Once standardized, immunological assays such as the ones we have developed here could enable a correlation to be developed, either by studying natural antibody levels from patients who have been exposed to *K. pneumoniae* or by studying vaccine-induced antibody levels. These correlates could then be used as an immunological endpoint to assist in the development and licensure of vaccines, which are urgently needed against this critical pathogen.

## MATERIALS AND METHODS

### Bacterial strains

Clinical isolates qualified for use in the OPAs can be seen in Table 1. The master stocks of the bacterial strains were stored at –80°C. Working stocks were grown by streaking flecks of frozen master stocks on a horse blood agar plate (Thermo Scientific Oxoid, UK) and incubating at 37°C/5% CO_2_ for 16–18 h. Using an inoculation loop, a single colony was mixed with 2% Luria Bertani (LB) broth (Sigma-Aldrich, UK) at 37°C/5% CO_2_ to an optical density at 600 nm (OD600) of 0.5 to 0.65, as this represented the log phase of growth. Working stocks of bacteria were stored at –80°C in 2% LB broth with 10% glycerol (Sigma-Aldrich, UK) and thawed on the day of use. To determine the optimal dilution of each serotype in the OPA, an optical density (OD1) assay was performed, where each bacterial isolate was serially diluted and spotted onto LB agar plates (1.5% agar), and dilutions that produced 75–150 colony-forming units (CFU) were selected for further optimization. Details of the evaluation of bacterial strains can be found in Table S1.

### CPS/OPS purification

Capsular and O antigen polysaccharides (CPS and OPS) were independently purified at Inventprise in a neutral minimal media supplemented with dextrose and trace vitamins and elements. Briefly, 1 mL of a seed stock was inoculated into a shake flask volume of 500 mL and incubated at 250 RPM, 37°C, for 5–18 h. Then, the culture was transferred into 7 L final volume in a BioFlo 320 (Eppendorf, Germany), where it was grown under the same conditions with 50% DO and additional dextrose supplementation for 10–15 h until the stationary phase. Culture was inactivated by acid hydrolysis and neutralized, and the cellular debris was removed by centrifugation for 30 min at 14,000 × *g*, 4°C. The supernatant was filtered with a 0.22-µm depth filter (Critical Process Filtration, USA) and stored at 4°C. CPS and OPS were isolated using the tangential flow filtration (TFF) cassette membranes for concentration and diafiltration using molecular weight cutoff (MWCO) based on polysaccharide size (Biomax; Millipore, USA). Additional separation was done using anion exchange chromatography in negative mode (Sartobind Q; Sartorius, Germany) and then concentrated with TFF of respective MWCO and 0.22 µm filtered for storage at −80°C. Polysaccharide concentration was determined by anthrone assay (34), protein impurities were assessed with Lowry assay, residual nucleic acid was measured by absorbance at 260 nm, and molecular size was determined using HPLC-SEC with refractive index (RI) on an Alliance e2695 system (Waters, USA) using (Shodex SB-804 and 805 HQ columns; Resonac, Japan). Identity was done using proton nuclear magnetic resonance imaging (^1^H-NMR).

### PS-CRM conjugates

Purified CPS and OPS of serotypes KL2, KL25, KL102, O1, and O5 were conjugated individually with rCRM197 using CDAP and EDC chemistry (35, 36). rCRM197 (cross-reactive material 197) is a detoxified diphtheria recombinant toxin produced by expression in *E. coli* cells (37). Conjugates were concentrated and diafiltered with TFF of appropriate MWCO. The pentavalent glycoconjugate vaccine is comprised of the five individual monovalent conjugates, formulated together with aluminum phosphate adjuvant. Three different formulations were produced as follows: (i) 2.5 µg PS per serotype, 125 µg Al^3+^ as aluminum phosphate; (ii) 2.5 µg PS per serotype, 250 µg Al^3+^ as aluminum phosphate; and (iii) 10 µg PS per serotype, 125 µg Al^3+^ as aluminum phosphate. Polysaccharide concentration was determined by anthrone assay, protein impurities were assessed with BCA assay, and molecular size was determined using HPLC-SEC with A280 and refractive index (RI) on an Alliance e2695 system (Waters, USA) using (Shodex SB-804 & 805 HQ columns; Resonac, Japan).

### HL-60 cells

Human promyelocytic leukemia-60 (HL-60) cells (ATCC Standards, USA) were stimulated with 0.8% N,N-dimethylformamide (DMF) (Sigma-Aldrich, UK) in RPMI 1640 [Thermo Fisher Scientific) supplemented with 10% FetalClone I serum (Hyclone, UK) and 1% L-glutamine (100×) (Thermo Fisher Scientific, UK). Cells were incubated at 37°C/5% CO_2_ for 5–6 days. For use in the assay, cells were counted and resuspended in opsonization buffer (1× HBSS [+Ca/Mg] with 10% FBS and 1% gelatin) to a final concentration of 1 × 10^7^ cells/mL, ensuring a viability exceeding 80%. A monthly assessment of the neutrophil-like phenotype of differentiated HL-60 cells was performed using flow cytometry, where cells were accepted for use if the upregulation of CD35 (complement receptor 1) was ≥55%, and the downregulation of CD71 (transferrin receptor 1) was ≤15%. Fluorescently labeled antibodies were mouse anti-human CD35 FITC (Bio-Rad, USA) and mouse anti-human CD71 PE (BD Biosciences, USA). The viability of differentiated HL-60 cells was assessed by annexin V-PI staining and was accepted if less than 20% of the cells were stained, indicative of apoptotic cells. MycoAlert mycoplasma detection kit (Lonza, Switzerland) was used monthly on the current working stock of cells to test for contamination. If cells tested positive for mycoplasma, the batch was discarded.

### Rabbit immunization and sera

New Zealand White rabbits were administered three doses of pentavalent vaccine (using CRM197 as a carrier protein) on days 0, 14, and 28, and sera were collected prior to each dose and on day 42. Each dose of the vaccine contained up to 10 µg of antigen per serotype including KL2, KL25, and KL102. Pooled rabbit sera from each of the three formulations were used for the precision assays of the appropriate OPAs. Aliquots of Hyperimmune globulins for intravenous use (H-IVIG) were kindly provided by Prof. Alan Cross (University of Maryland School of Medicine, Baltimore, MD, USA). H-IVIG constituted pooled plasma from donors immunized with a 24-valent *K. pneumoniae* polysaccharide vaccine containing 50 µg of CPS antigen per dose (26), including the five serotypes of interest in this manuscript. H-IVIG was used during the strain selection process, the precision of KL15 and KL62, and the specificity and linearity of all OPAs. Human normal subcutaneous immunoglobulin (SCIg) (Cutaquig, 165 mg/mL) obtained from Great Ormond Street Hospital (GOSH) was used as a quality control (QC) for all assays.

### Opsonophagocytic killing assay (OPA)

The OPA assay was adapted from the validated UCL *Streptococcus pneumoniae* MOPA (38) and is now qualified for capsular *K. pneumoniae* serotypes since its first iteration as an LPS *K. pneumoniae* OPA, which has been previously described (23). Briefly, heat-inactivated serum samples were serially diluted 3-fold in opsonization buffer in duplicate in a 96-well plate. Working stocks of frozen bacterial strains were thawed as required, washed twice in opsonization buffer, and pre-diluted to an optimum dilution that yielded a final colony count between 75 and 150 CFU (previously determined by OD1 assay). Diluted bacteria were added to every well, and the plate was incubated on an orbital shaker at 700 rpm for 30 min at RT. Baby rabbit complement (BRC) (Pel-Freez, USA) was thawed on ice and diluted in OPS buffer to a pre-determined optimal dilution (4%–8.75% final, depending on serotype) that allowed for killing in the assay while maintaining low levels of non-specific killing (NSK). Diluted BRC and differentiated HL-60 cells at 1 × 10^7^/mL were added to every well, and the plate was incubated on an orbital shaker at 750 rpm for 45 min at 37°C/5% CO_2_. Plates were placed on ice for 20 min, and each well was spotted onto Luria Bertani (LB) agar plates (1.5% agar). Once dried, an LB overlay (0.75% agar) containing 2,3,5-triphenyl tetrazolium chloride (TTC) (0.1%) was added to better distinguish individual colonies. Plates were inverted and incubated at 37°C/5% CO_2_ for 16–18 h before counting the surviving colony forming units (CFU) with an automated colony counter and ProtoCOL software (Synbiosis).

### Specificity

Specificity in the OPA was performed as a competition study, where H-IVIG was pre-adsorbed with either homologous or heterologous purified polysaccharide antigens. Antigens were diluted to 1 µg/mL and pre-adsorbed with sera for >60 min at room temperature (RT) before continuing the assay protocol. % inhibition was calculated as 100 – (OI with competitor/OI without competitor) × 100.

### Statistics

Statistical analysis and graphing were performed using GraphPad Prism version 10.0.2 (GraphPad, San Diego, CA) and Microsoft Excel. %NSK was defined as 1 – [CFU (control B)/CFU (control A)] × 100, where control A contained bacteria, HL-60 cells, and heat-inactivated complement, and control B contained bacteria, HL-60 cells, and active complement. The percentage killing at each dilution of a sample was calculated as [CFU (control B) – CFU (sample)]/CFU (control B)] × 100, and the dilution of the sample resulting in 50% killing was calculated as the opsonic index (OI) using Opsotiter software version 3 (Bacterial Respiratory Pathogen Reference Laboratory, University of Alabama at Birmingham, USA). Linear regression analysis of the OPAs was performed to assess linearity, and the Pearson’s correlation coefficient was calculated using GraphPad Prism version 10.0.2.

## SUPPLEMENTAL MATERIAL

Table S1 msphere.00176-25-s0001.docx Evaluation of bacterial strains.

ASM does not own the copyrights to Supplemental Material that may be linked to, or accessed through, an article. The authors have granted ASM a non-exclusive, world-wide license to publish the Supplemental Material files. Please contact the corresponding author directly for reuse.
