# Supplementary material for: The development of functional opsonophagocytic assays to evaluate antibody responses to Klebsiella pneumoniae capsular antigens
Source: mSphere. 2025 Jun 12;10(7):e00176-25. doi: 10.1128/msphere.00176-25 (PMC12306171; doi:10.1128/msphere.00176-25)
Supplement: Table S1 — Evaluation of bacterial strains. [file msphere.00176-25-s0001.docx]

| **Supplementary Table 1.** Evaluation of bacterial strains | | | |  |  |  |  |
| --- | --- | --- | --- | --- | --- | --- | --- |
| **Serotype** | **No. of strains evaluated by OD1** | **No. excluded due to NSK >35%** | **No. excluded due to CFU < 50** | **No. of strains evaluated by OD2** | **No. excluded due to IK** | **No. evaluated for specificity** |  |
| KL2 | 27 | 1 | 1 | 16 | 0 | 7 |  |
| KL15 | 8 | 1 | 0 | 7 | 1 | 6 |  |
| KL25 | 11 | 1 | 1 | 4 | 1 | 3 |  |
| KL62 | 11 | 1 | 0 | 10 | 1 | 5 |  |
| KL102 | 19 | 12 | 2 | 5 | 0 | 2 |  |
| Strains were excluded after OD1 if the NSK was >35% or if the CFU failed to reach at least 50. Many strains were then run with plasma (H-IVIG) by OD2, although not every strain that passed OD1 was necessarily run on OD2. Susceptible strains with an intermediate OI were then screened in a specificity assay and the final strain selected for qualification was the strain with the highest homologous inhibition and lowest heterologous inhibition. OD1= Optical Density 1 Assay, OD2= Optical Density 2 Assay, NSK = Non-Specific Killing, CFU = Colony-Forming Units, IK = Incomplete Killing (Max Killing <70%), H-IVIG = Hyper-Immune Globulins for Intravenous use. | | | | | | |  |
|  |  |  |  |  |  |  |  |
|  |  |  |  |  |  |  |  |
|  |  |  |  |  |  |  |  |
|  |  |  |  |  |  |  |  |
|  |  |  |  |  |  |  |  |
|  |  |  |  |  |  |  |  |
